# Attitudes Toward Artificial Intelligence Among Polish Dentists: A Cross-Sectional Survey

**DOI:** 10.3390/diagnostics16142271

**Published:** 2026-07-21

**Authors:** Michalina Nowakowska, Leszek Szalewski

**Affiliations:** 1 Radus Stomatologia, ul.Bóżnicza 1/208, 61-651 Poznan, Poland; 2 Digital Dentistry Lab, Department of Dental and Maxillofacial Radiodiagnostics, Medical University of Lublin, ul. Chodzki 6, 20-093 Lublin, Poland; leszek.szalewski@umlub.pl

**Keywords:** artificial intelligence, dentistry, dental practice, questionnaire, technology acceptance

## Abstract

**Background:** Artificial intelligence (AI) is increasingly being integrated into modern dental practice, particularly in diagnostics, radiographic analysis, treatment planning, and practice management. Despite the rapid advancement of AI-based technologies, evidence regarding dentists’ attitudes toward AI in Central and Eastern Europe remains limited. This study aimed to evaluate Polish dentists’ attitudes toward artificial intelligence in contemporary dental practice and to investigate differences in AI acceptance according to sex, age group, and dental specialty. **Materials and Methods:** A cross-sectional online questionnaire-based study was conducted among licensed dentists practicing in Poland. An anonymous questionnaire comprising 15 attitude statements rated on a five-point Likert scale was distributed through professional social media groups. The survey assessed attitudes toward the use of AI in clinical, diagnostic, and administrative aspects of dentistry. Statistical analyses were performed using Statistica 16.0. Group comparisons were conducted using the Mann–Whitney U test and Kruskal–Wallis test with Benjamini–Hochberg false discovery rate correction. Internal consistency of the questionnaire was assessed using Cronbach’s alpha coefficient. **Results:** A total of 183 completed questionnaires were included in the analysis. The internal consistency of the questionnaire was high (Cronbach’s α = 0.900). The overall acceptance of AI was moderate (mean score: 3.18 ± 0.73). The highest levels of agreement were observed for the perceived potential of AI to improve dental practice management (mean = 4.05) and for general openness toward AI implementation in dentistry (mean = 4.05). Respondents also expressed a high willingness to use AI for generating clinical documentation (mean = 3.69). In contrast, the lowest acceptance was observed for statements suggesting that AI could replace dentists (mean: 1.36). Within the study sample, men demonstrated significantly higher overall AI acceptance than women (*p* < 0.001). Significant differences were also observed between age groups and dental specialties. Orthodontists demonstrated the highest AI acceptance among the surveyed specialties; however, these findings should be interpreted as exploratory because of unequal subgroup sizes. After FDR correction, significant differences between age groups remained only for selected questionnaire items. **Conclusions:** Within the limitations of this convenience sample, the findings suggest that Polish dentists generally perceive artificial intelligence as a supportive tool rather than a replacement for clinicians. Acceptance was greatest for administrative and organizational applications of AI, whereas autonomous clinical decision-making received substantially lower support. These findings suggest that the successful implementation of AI in dentistry should prioritize assistive technologies that enhance clinical workflows while preserving the central role of the dentist in patient care.

## 1. Introduction

The term artificial intelligence (AI) was first introduced in 1950 and refers to the ability of machines to perform tasks that have traditionally required human intelligence. One of the greatest advantages of AI systems is their capacity to solve complex problems, particularly those that are difficult or impossible to address using conventional methods [1]. The application of AI algorithms to support or partially replace human involvement in data and image analysis, as well as clinical decision-making, has become both a major challenge and a rapidly evolving area of modern medicine [2].

Over the past two decades, AI-based technologies have developed rapidly in dentistry. They have become an integral component of contemporary dental practice and are increasingly used in diagnostics, dental radiology, orthodontics, prosthodontics, and treatment planning [3]. Beyond clinical applications, AI is also being integrated into administrative tasks and dental practice management. Dental support staff—including practice managers, dental assistants, and administrative personnel—play a crucial role in the organization and daily operation of dental clinics. Routine tasks such as appointment scheduling, patient communication, billing, and administrative documentation are increasingly being supported or automated by AI-based applications [4]. AI-powered virtual dental assistants have also become commercially available. These technologies enable the automation of repetitive administrative tasks with high consistency and a potentially lower risk of error, thereby reducing the workload of dental personnel [5].

A growing body of evidence suggests that AI has the potential to improve the safety, efficiency, and precision of healthcare services. At the same time, the increasing adoption of AI raises important questions regarding trust, ethical considerations, legal responsibility, and the future organization of dental practice [3]. To the best of our knowledge, evidence regarding dentists’ attitudes toward artificial intelligence in Central and Eastern Europe remains limited, particularly in countries undergoing rapid digital transformation, such as Poland.

This study aimed to evaluate Polish dentists’ attitudes toward the use of artificial intelligence in contemporary dental practice and to investigate differences in AI acceptance according to sex, age group, and dental specialty.

## 2. Materials and Methods

### 2.1. Study Design and Participants

This cross-sectional questionnaire-based study was conducted using an anonymous online survey created in Google Forms. The questionnaire consisted of 18 items (Appendix A), including three demographic questions and fifteen statements assessing attitudes toward artificial intelligence in dentistry. Responses to the attitude statements were recorded using a five-point Likert scale ranging from 1 (“strongly disagree”) to 5 (“strongly agree”).

The questionnaire was specifically developed for this study based on a review of the current literature on the application of artificial intelligence in dentistry, as well as the authors’ clinical experience and research objectives. Before distribution, the preliminary version of the questionnaire underwent pilot testing among dental interns to evaluate the clarity, comprehensibility, and readability of the survey items. Based on the feedback received, minor linguistic modifications were introduced. The revised questionnaire was subsequently reviewed by a panel of experienced academic dentists from the Medical University of Lublin to assess the relevance and content validity of the survey items before the final version was distributed.

Participation was voluntary and restricted to licensed dentists practicing in Poland. The questionnaire was distributed through three large Facebook groups dedicated to Polish dentists (“Dentyści”, “DENTYŚCI–Przypadki, Kursy, Dyskusje”, and “ML Stomatolog”). Participant recruitment and data collection were conducted between 1 January and 1 April 2026. This exploratory cross-sectional study was based on a convenience sample of voluntarily participating dentists recruited through professional social media groups. No formal sample size calculation was performed because of the exploratory nature of the study. A total of 184 responses were received, of which one incomplete questionnaire was excluded, resulting in a final study sample of 183 respondents.

### 2.2. Ethical Considerations

Participation in the study was voluntary and anonymous. Before accessing the questionnaire, all participants were presented with an introductory information page describing the purpose of the study, the voluntary nature of participation, the anonymous handling of the collected data, their intended use for scientific purposes, the potential benefits and minimal risks associated with participation, and the estimated time required to complete the questionnaire (approximately 3–5 min). Participants were informed that they could withdraw from the survey at any time before submitting their responses without providing a reason.

Completion and submission of the questionnaire were considered to constitute informed consent to participate in the study.

According to the institutional practice governing anonymous questionnaire-based research, formal approval from the Bioethics Committee was not required because the study involved a fully anonymous survey and did not collect identifiable personal data or sensitive health information. The study was conducted in accordance with the ethical principles of the Declaration of Helsinki.

### 2.3. Statistical Analysis

Statistical analyses were performed using Statistica 16.0 (StatSoft, Kraków, Poland). The normality of data distribution was assessed using the Shapiro–Wilk test. The internal consistency of the questionnaire was evaluated using Cronbach’s alpha coefficient.

**Figure 1:**
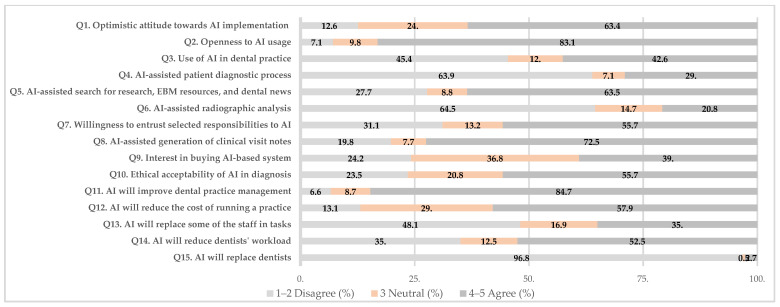
Distribution of responses to the questionnaire items. Values of 1–2 indicate disagreement or strong disagreement, 3 indicates a neutral response, and 4–5 indicate agreement or strong agreement.

The Total AI Acceptance Score was calculated as the arithmetic mean of 13 attitude items (Figure 1: Q1–Q15), excluding Q13 (“AI will replace some of the staff in tasks”) and Q15 (“AI will replace dentists”). These two items were intentionally excluded because they assessed respondents’ expectations regarding the potential future role of AI rather than their acceptance of AI as a supportive technology in contemporary dental practice. No reverse coding was applied. The excluded items were analyzed separately as individual questionnaire items. The resulting score ranged from 1 to 5, with higher values indicating greater acceptance of artificial intelligence in dentistry.

Comparisons between women and men were performed using the Mann–Whitney U test, whereas differences between age groups and dental specialties were analyzed using the Kruskal–Wallis test. To account for multiple comparisons and reduce the likelihood of false-positive findings, *p*-values were adjusted using the Benjamini–Hochberg false discovery rate (FDR) procedure, which controls the expected proportion of false-positive findings among all statistically significant results. When appropriate, post hoc pairwise comparisons were performed using the Holm correction. Statistical significance was established at q < 0.05.

## 3. Results

A total of 183 questionnaires were included in the analysis. The study sample consisted predominantly of women (148; 80.9%) and participants aged 30–40 years (87; 47.5%) (Table 1). The 15-item questionnaire demonstrated high internal consistency (Cronbach’s α = 0.900). The mean Total AI Acceptance Score was 3.18 ± 0.73 (median = 3.27).

The highest levels of agreement were observed for statements indicating that AI-based systems could improve dental practice management (Q11; mean = 4.05; 84.7% of responses rated 4–5), general openness toward AI implementation in dentistry (Q2; mean = 4.05; 83.1%), and willingness to use AI for generating clinical documentation (Q8; mean = 3.69; 72.5%). In contrast, the lowest level of agreement was observed for the statement suggesting that AI could replace dentists (Q15; mean = 1.36; 2.7% of responses rated 4–5). The distribution of responses to all questionnaire items is presented in Figure 1.

Within the study sample, men demonstrated significantly higher overall AI acceptance than women (3.58 vs. 3.08; U = 1544; *p* < 0.001; q = 0.002). After FDR correction, these differences remained statistically significant for 10 of the 15 questionnaire items, including general attitudes toward AI, openness to its use, implementation in clinical practice, perceived ethical acceptability, and the belief that AI could reduce the operating costs of dental practices. Detailed results of the sex-based comparisons are presented in Table 2.

Age-stratified analysis revealed statistically significant differences after FDR correction for the use of AI in clinical practice (Q3), AI-assisted diagnostic process (Q4), and the perceived ethical acceptability of AI in diagnostics (Q10), with the highest scores observed among respondents aged 40–50 years. Detailed results of the age-group comparisons are presented in Table 3.

The exploratory analysis across dental specialties demonstrated significant differences in the use of AI in clinical practice, AI-assisted radiographic analysis, interest in purchasing AI-based equipment, and the perceived potential of AI to reduce practice costs. Orthodontists consistently demonstrated the highest mean scores across the significant questionnaire items. However, because several specialty groups were represented by relatively small sample sizes, these findings should be considered exploratory and be interpreted with caution. Detailed results according to dental specialty are presented in Table 4.

## 4. Discussion

The present study demonstrated a moderate level of acceptance of artificial intelligence among Polish dentists. The highest acceptance was observed for administrative and organizational applications of AI, particularly those supporting practice management and the generation of clinical documentation. In contrast, respondents expressed considerably lower acceptance of AI replacing dentists or making autonomous clinical decisions. These findings suggest that Polish dentists currently perceive AI primarily as a supportive tool rather than a substitute for professional clinical judgment.

These findings are consistent with previous reports demonstrating the increasing adoption of artificial intelligence in dentistry, particularly in diagnostics, treatment planning, radiographic analysis, and practice management [6]. Previous studies have reported that approximately 35% of dentists worldwide have implemented AI-based systems in their clinical practice. In North America, approximately 18% of dental practices currently use AI, primarily for diagnostic support, medical imaging, and practice management. In Europe, however, the implementation of AI-based systems in dental practices is gradual, primarily due to legal regulations [7]. Similarly, the respondents in our study expressed the greatest acceptance of AI applications for generating clinical documentation and supporting dental practice management.

In the present study, orthodontists demonstrated the highest level of AI acceptance among the surveyed specialties. This observation is consistent with previous studies reporting that orthodontics is among the dental specialties with the greatest implementation of AI-based technologies [8]. Other specialties frequently highlighted in the literature include endodontics and periodontology [8,9,10]. One possible explanation for this finding is the widespread integration of digital technologies into contemporary orthodontic practice. Orthodontists routinely use digital workflows, including cephalometric analysis, three-dimensional imaging, digital treatment planning, aligner therapy, treatment simulation software, and other AI-assisted diagnostic and planning tools. Greater day-to-day exposure to these technologies may contribute to greater familiarity with and acceptance of AI in clinical practice. However, because several specialty groups were represented by relatively small numbers of participants, these findings should be considered exploratory and interpreted with caution. In particular, the periodontology subgroup included only five respondents, substantially limiting the reliability of comparisons involving this specialty. Therefore, confirmation in larger and more balanced study populations is warranted. In our study, men demonstrated significantly higher overall acceptance of AI compared to women, particularly in attitude toward AI, openness to its use, implementation in clinical practice, perceived ethical compatibility, and the belief that AI can reduce practice costs. In the study by Sarhan et al., men were more likely than women to believe that the use of AI is merely a trend but were more convinced that AI-based systems could replace dentists [9]. Similarly, Hegde et al. [11] reported that more women perceived AI more positively as a clinical support tool and differed in comparing their clinical assessment to AI-based systems, whereas men were more likely to use AI-based applications. However, the observed sex differences should be interpreted with caution. Because of the cross-sectional design of the study, no causal inferences can be drawn, and the markedly smaller number of male participants compared with female participants limits the generalizability of these findings. Therefore, these observations should be considered exploratory and require confirmation in larger, more balanced populations.

In the present study, respondents aged 40–50 years demonstrated higher scores for selected aspects of AI acceptance, including its use in clinical practice, AI-assisted diagnostics, and perceived ethical compatibility. However, no significant differences in the overall AI Acceptance Score between age groups remained after correction for multiple comparisons. Therefore, these findings should be interpreted as relating to specific aspects of AI acceptance rather than to overall attitudes toward AI. These findings differ from those reported by Sarhan et al. [9], who observed that the youngest age group (20–25 years) were more likely to use AI-based systems, had more optimistic attitudes toward AI-assisted dentistry, and participated more frequently in courses and webinars on this topic [9]. In another study, respondents aged 35 (+/−14) were more aware of the possibility of implementing AI-assisted systems in dental everyday practice compared to those aged 28 (+/−6) [11]. These discrepancies may reflect differences in the availability of AI technologies, levels of digitalization, and the pace of technological development across countries.

In our study, respondents were least likely to agree on the possibility of AI replacing dentists. The potential of AI to replace dentists remains one of the most controversial aspects of AI implementation in dentistry. In a study conducted in South Korea, the majority of dental students and dentists did not believe that AI could replace their jobs (64.2% and 71.9%, respectively), despite positively assessing its potential and usefulness in daily practice. Most respondents agreed that AI was unlikely to replace all professional roles but expected it to automate relatively simple and repetitive tasks [12]. In a study conducted in Egypt, positive perceptions of AI’s role in a specialty development and replacement potential were significantly associated with lower levels of education; holders of bachelor’s (BD) and master’s (MSc) degrees were significantly more likely to share these views than those with a doctor of medicine degree [9]. Although many practitioners recognize the potential benefits of AI in everyday dental practice, its clinical implementation remains limited by educational, ethical, legal, and practical barriers. Dentists provide personalized care, build trust, and develop relationships with patients, often based on empathy and mutual understanding. Despite its remarkable capabilities, AI cannot replace essential human attributes such as empathy, ethical judgment, and interpersonal communication, all of which remain fundamental to high-quality dental care.

Several limitations of this study should be considered when interpreting the findings. First, this was a cross-sectional study based on a convenience sample recruited through professional Facebook groups and no formal sample size calculation was performed. Therefore, the study population may not be fully representative of all dentists practicing in Poland, and self-selection bias cannot be excluded, as dentists with a greater interest in digital technologies and artificial intelligence may have been more likely to participate. In addition, because the survey was distributed through social media, the response rate could not be determined. Consequently, the observed level of AI acceptance may be higher than that in the broader population of Polish dentists, limiting the generalizability of the findings.

Second, the questionnaire was developed specifically for this study. Although it underwent pilot testing among dental interns and expert review by experienced academic dentists and demonstrated high internal consistency, it was not subjected to a formal psychometric validation process using standardized validation procedures.

Third, because the survey was fully anonymous and no personal identifiers were collected, it was not possible to verify whether duplicate responses had been submitted. Although there was no indication that this occurred, this possibility cannot be completely excluded.

Finally, the unequal distribution of participants across sex, age groups, and dental specialties, together with the relatively small sample sizes of several specialty groups, limits the generalizability of subgroup analyses. Therefore, these findings should be regarded as exploratory. Furthermore, the cross-sectional design reflects attitudes at a single point in time and does not allow assessment of changes in perceptions as AI technologies continue to evolve.

The rapid evolution of artificial intelligence technologies may influence dentists’ opinions and experiences, meaning that attitudes toward AI in dentistry may continue to change alongside increasing clinical implementation and technological development.

Overall, the findings indicate that dentists perceive artificial intelligence primarily as a supportive tool rather than a replacement for clinical expertise. The highest level of acceptance was observed for administrative and organizational applications, such as medical documentation generation and improving the efficiency of dental practice management. These observations suggest that the initial integration of AI into dentistry may be most successful in areas that reduce workload and optimize everyday clinical routines.

The relatively lower acceptance of autonomous AI-based clinical decision-making highlights the continued importance of the dentist’s professional judgment, ethical responsibility, and interpersonal communication with patients. Therefore, future implementation of AI systems in dentistry should focus on developing assistive technologies that complement, rather than replace, the clinician’s role.

The observed differences between age groups and specialties also suggest that targeted educational programs and practical training are likely to be essential to improve familiarity with AI-based tools and facilitate their responsible adoption in everyday dental practice. Furthermore, increasing awareness of the benefits and limitations of AI may help reduce uncertainty and improve clinicians’ confidence in using these technologies.

Future studies should include longitudinal, multicenter investigations evaluating the real-world implementation of artificial intelligence in dental practice. Further investigations involving larger and more balanced groups of specialists may provide a more comprehensive understanding of factors influencing AI acceptance among dentists.

In addition, future studies should assess the effectiveness of educational interventions and training programs designed to improve clinicians’ knowledge and confidence regarding AI-based technologies. Future research should also investigate patients’ perceptions on the use of artificial intelligence in dentistry, particularly in relation to trust, communication, and ethical concerns.

Finally, continued clinical validation together with the development of clear ethical and legal frameworks will be essential to ensure the safe, effective, and responsible integration of artificial intelligence into contemporary dental practice.

## 5. Conclusions

Overall, the findings indicate a moderate level of acceptance of artificial intelligence (AI) as a tool to support, rather than replace, clinical practice. Within the limitations of this convenience sample, Polish dentists appeared to be most receptive to AI applications for administrative and organizational tasks, whereas substantially lower acceptance was observed for AI systems intended to replace dentists or perform autonomous clinical decision-making.

These findings suggest that the successful integration of AI into dentistry will depend primarily on the development of assistive technologies that complement, rather than replace, the clinician’s expertise, professional judgment, and patient-centered decision-making. The differences observed among dental specialties should be regarded as exploratory because several specialty groups were represented by relatively small numbers of participants. Consequently, these findings should be confirmed in larger studies involving more balanced populations of dental specialists.

These findings may help guide the implementation of AI technologies in dental practice and support the development of educational initiatives aimed at improving dentists’ familiarity with AI-based tools.

## Figures and Tables

**Table 1:** Characteristics of the study sample.

Variable	Category	*n* (%)
Sex	Women	148 (80.9%)
	Men	35 (19.1%)
Age	20–30 years old	52 (28.4%)
	30–40 years old	87 (47.5%)
	40–50 years old	34 (18.6%)
	50–60 years old	10 (5.5%)
Dental specialties	Restorative dentistry	74 (40.7%)
	Prosthetics	15 (8.2%)
	Orthodontics	23 (12.6%)
	Dental/maxillo-facial surgery	18 (9.9%)
	Pedodontics	22 (12.1%)
	Endodontics	25 (13.7%)
	Periodontology	5 (2.7%)
	No data	1 (0.5%)

**Table 2:** Sex-based comparisons of questionnaire responses after FDR correction.

	Women: Mean (Me)	Men: Mean (Me)	*p*	q_FDR
Optimistic attitude towards AI implementation	3.64 (Me = 4)	4.23 (Me = 5)	<0.001	0.003
Openness to AI usage	3.96 (Me = 4)	4.46 (Me = 5)	<0.001	0.003
Use of AI in dental practice	2.78 (Me = 2)	3.54 (Me = 4)	0.003	0.007
AI-assisted patient diagnostic process	2.35 (Me = 2)	3.09 (Me = 4)	0.019	0.030
Interest in buying AI-based system	3.08 (Me = 3)	3.71 (Me = 4)	0.005	0.012
Ethical acceptability of AI in diagnostic process	3.28 (Me = 4)	3.71 (Me = 4)	0.018	0.030
AI will improve dental practice management	3.97 (Me = 4)	4.43 (Me = 5)	<0.001	0.003
AI will reduce the cost of running a practice	3.49 (Me = 4)	4.14 (Me = 4)	<0.001	0.002
AI will replace some of the staff in tasks	2.76 (Me = 2)	3.31 (Me = 4)	0.026	0.038
AI will reduce dentists’ workload	3.10 (Me = 3)	3.71 (Me = 4)	0.006	0.012
Total AI Acceptance Score (average of 13 items)	3.08 (Me = 3)	3.58 (Me = 4)	<0.001	0.002

Comparisons were performed using the Mann–Whitney U test. Statistical significance was defined as qFDR < 0.05.

**Table 3:** Comparison of questionnaire responses among age groups.

	20–30 y.o.	30–40 y.o.	40–50 y.o.	50–60 y.o.	H	*p*	q_FDR	Relevant After FDR
Use of AI in dental practice	2.54 (Me = 2)	2.84 (Me = 3)	3.62 (Me = 4)	3.30 (Me = 4)	14.41	0.002	0.038	yes
AI-assisted patient diagnostic process	2.13 (Me = 2)	2.41 (Me = 2)	3.15 (Me = 4)	2.80 (Me = 2)	11.80	0.008	0.043	yes
Use of AI in diagnostics is ethical	3.19 (Me = 3)	3.22 (Me = 4)	3.94 (Me = 4)	3.60 (Me = 4)	12.26	0.007	0.043	yes
Total AI Acceptance Score (average of 13 items)	3.05 (Me = 3)	3.12 (Me = 3)	3.51 (Me = 4)	3.28 (Me = 3)	8.67	0.034	0.136	no

Comparisons were performed using the Kruskal–Wallis test. Statistical significance was defined as qFDR < 0.05.

**Table 4:** Comparison of questionnaire responses among dental specialties.

	Restorative Dentistry	Prosthetics	Orthodontics	Surgery	Pedodontics	Endodontics	Perio	H	*p*	q_FDR
Use of AI in dental practice	2.65	2.87	4.04	2.67	3.09	2.60	3.60	22.65	< 0.001	0.006
AI-assisted patient diagnostic process	2.24	2.20	3.43	2.39	2.86	2.40	2.00	17.53	0.008	0.030
AI-assisted radiographic analysis	2.18	2.20	3.48	2.11	2.55	2.04	2.40	22.82	< 0.001	0.006
Interest in buying AI-based system	3.12	3.07	3.96	3.06	3.14	2.92	3.60	15.28	0.018	0.048
AI will reduce the cost of running a practice	3.39	3.40	4.26	3.22	3.68	4.00	4.00	22.18	0.001	0.006
Total AI Acceptance Score (average of 13 items)	3.09	3.12	3.66	2.97	3.25	3.07	3.36	15.51	0.017	0.048

Comparisons were performed using the Kruskal–Wallis test. Because of the unequal subgroup sizes, the results should be interpreted as exploratory. Statistical significance was defined as qFDR < 0.05.

## Data Availability

The data that support the findings of this study are available from the corresponding author upon reasonable request.

## Abbreviations

The following abbreviations are used in this manuscript:

*n*	number of respondents
%	percentage of respondents
SD	standard deviation
Me	median
IQR	interquartile range
qFDR	*p*-values adjusted using the Benjamini–Hochberg false discovery rate correction
H	Kruskal–Wallis test statistic

## Appendix A. Questionnaire

1. Please provide your gender.

- man	- woman

2. Please indicate your age group.

- 50–60 years old	- 20–30 years old
- 60+ years old	- 30–40 years old
	- 40–50 years old

3. Please indicate your main field of dental practice.

- orthodontics	- restorative dentistry
- periodontology	- endodontics
- pedodontics	- dental surgery
- maxillo-facial surgery	- prosthodontics

Q1. I have an optimistic attitude towards AI implementation in dentistry.

- I strongly agree
- I agree
- I have no opinion
- I disagree
- I strongly disagree

Q2. I am open to using AI in dentistry.

- I strongly agree
- I agree
- I have no opinion
- I disagree
- I strongly disagree

Q3. I use AI-based systems in my dental practice.

- I strongly agree
- I agree
- I have no opinion
- I disagree
- I strongly disagree

Q4. I use AI-assisted tools in the diagnostic process.

- I strongly agree
- I agree
- I have no opinion
- I disagree
- I strongly disagree

Q5. I use AI-assisted tools to search for new research, evidence-based medicine (EBM) resources, and dental news.

- I strongly agree
- I agree
- I have no opinion
- I disagree
- I strongly disagree

Q6. I use AI-assisted tools for radiographic analysis.

- I strongly agree
- I agree
- I have no opinion
- I disagree
- I strongly disagree

Q7. I would be willing to entrust some of my work responsibilities to AI-based systems.

- I strongly agree
- I agree
- I have no opinion
- I disagree
- I strongly disagree

Q8. I use or would be willing to use AI-assisted tools for generating clinical visit notes.

- I strongly agree
- I agree
- I have no opinion
- I disagree
- I strongly disagree

Q9. I am interested in buying AI-based dental equipment.

- I strongly agree
- I agree
- I have no opinion
- I disagree
- I strongly disagree

Q10. I believe that the use of AI in the diagnostic process is consistent with medical ethics.

- I strongly agree
- I agree
- I have no opinion
- I disagree
- I strongly disagree

Q11. I believe that AI-based systems can improve the functioning of dental practices.

- I strongly agree
- I agree
- I have no opinion
- I disagree
- I strongly disagree

Q12. I believe that AI-based systems can reduce the costs of running a dental practice.

- I strongly agree
- I agree
- I have no opinion
- I disagree
- I strongly disagree

Q13. I believe that artificial intelligence can replace some dental staff in selected tasks.

- I strongly agree
- I agree
- I have no opinion
- I disagree
- I strongly disagree

Q14. I believe that AI can reduce dentists’ workload.

- I strongly agree
- I agree
- I have no opinion
- I disagree
- I strongly disagree

Q15. I believe that AI can replace dentists.

- I strongly agree
- I agree
- I have no opinion
- I disagree
- I strongly disagree
